# Draft Genome Sequences of Multiple *Streptomyces* Isolates from Arizona

**DOI:** 10.1128/MRA.00208-21

**Published:** 2021-07-01

**Authors:** David A. Baltrus, Aaron White, Caitlin Smith, Meara Clark

**Affiliations:** a School of Plant Sciences, University of Arizona, Tucson, Arizona, USA; b School of Animal and Comparative Biomedical Sciences, University of Arizona, Tucson, Arizona, USA; Loyola University Chicago

## Abstract

*Streptomyces* strains are bacteria that are well known for their distinctive physiology, behaviors, and ecology, as well as for being prodigious producers of diverse antibiotics. Here, we report draft genome sequences for eight *Streptomyces* strains that were isolated from multiple sky islands in Arizona and sequenced using an Oxford Nanopore Technologies Flongle adapter and MinION system.

## ANNOUNCEMENT

*Streptomyces* strains are well known for their ability to produce antibiotics and other secondary metabolites but are also emerging systems for studying diversity in bacterial lifestyles and behaviors, as well as for investigating bacterial patterns of biogeography and strain dispersal (1, 2). Therefore, the publication of closely related genomes of bacterial strains isolated within this distinct biogeographical framework could bolster our understanding of bacterial dispersal and endemism while potentially shedding light on the genetic bases underlying interesting variations in bacterial physiology, behavior, and antibiotic production.

The *Streptomyces* strains reported here were originally isolated from multiple sites in Arizona categorized as “sky islands” (3). For isolation, approximately 2 tablespoons of topsoil was sampled from grassy locations at three distinct sites, as listed in Table 1. Approximately 500-mg subsamples of the soil were then resuspended in 10 ml of distilled water, vortex mixed until the soil was well dispersed, and allowed to sit undisturbed until particulates settled (∼10 min). At that point, 500 μl was pipetted from the top of each sample onto glycerol-arginine plates supplemented with 300 μg/ml of cycloheximide and 50 μg/ml of rose bengal (as described in reference 2), which were then covered with Parafilm and incubated at 28°C for up to 2 weeks. Colonies displaying morphology resembling that of *Streptomyces* strains were sampled as they arose, with spores from each colony being streaked to new individual glycerol-arginine plates. After two or three isolations per sample, spore preparations were spread onto single glycerol-arginine plates, and spores were sampled for long-term storage in glycerol at −80°C. Immediately prior to preparation of spore stocks for long-term storage, each strain was grown in 2 ml of lysogeny broth (LB) liquid medium, and genomic DNA was isolated using a Wizard kit (Promega) with added lysozyme steps, as suggested in the Wizard kit protocol. PCR of the *rpoB* locus was performed for each sample using the primers and conditions described in reference 2. Amplified fragments arising from the PCRs were sequenced by Eton Biosciences (San Diego, CA, USA) using the primer sets described in reference 2. Sequence fragments were trimmed manually for quality (excluding Ns and ambiguous bases) and aligned using ClustalW v2.1 (4), and phylogenetic relationships between strains were inferred using MrBayes v3.2.7 (5).

**TABLE 1:** Provenance, sequencing, assembly, and genome characteristics of strains reported in this study

Strain	No. of reads	Read *N*_50_ (kb)	Total amount of DNA sequenced (Mb)	Barcode	SRA accession no.	Total assembly size (bp)	Contig sizes (bp)	GC content (%)	GenBank accession no.	Location of isolation	Isolation time
*Streptomyces* sp. strain AD2-2	129,616	29,349	1,848.25	NA^*a*^	SRR13716330	10,235,239	9,974,254, 133,008, 127,977	70.4	JAFELC000000000.2	31°54′48.98″N, 109°16′6.82″W	April 2017
*Streptomyces* sp. strain AD2-3	92,000	31,979	1,766.47	NA	SRR13716331	10,106,979	9,835,689, 140,603, 130,687	70.4	JAFELB000000000.2	31°54′48.98″N, 109°16′6.82″W	April 2017
*Streptomyces* sp. strain BD1-1	77,099	23,600	903.11	2	SRR13716332	9,937,615	9,786,967, 150,648	70.2	JAFELA000000000.2	31°54′48.98″N, 109°16′6.82″W	April 2017
*Streptomyces* sp. strain P3	56,820	21,814	549.19	3	SRR13716335	9,992,787	9,875,616, 117,171	70.4	JAFEKX000000000.2	32°27′20.34″N, 110°46′49.97″W	November 2016
*Streptomyces* sp. strain ML1-2	50,546	20,305	580.82	4	SRR13716333	7,231,652	2,167,131, 5,016,213, 48,308	72	JAFEKZ000000000.2	32°26′54.92″N, 110°46′51.59″W	November 2016
*Streptomyces* sp. strain ML2-9	77,878	21,918	719.11	5	SRR13716334	7,454,516	7,127,956, 326,560	72.5	JAFEKY000000000.2	32°26′54.92″N, 110°46′51.59″W	November 2016
*Streptomyces* sp. strain XL-6	60,483	22,286	724.48	6	SRR13716336	7,569,027	7,350,807, 136,208, 82,012	72.4	JAFEKW000000000.2	32°26′54.92″N, 110°46′51.59″W	November 2016
*Streptomyces* sp. strain XL-10	55,371	17,615	606.52	7	SRR13716337	7,284,057	7,078,668, 205,389	72.3	JAFEKV000000000.2	32°26′54.92″N, 110°46′51.59″W	November 2016

a NA, not applicable.

From the resulting phylogenies, eight strains in total, consisting of four strains each from two distinct phylogenetic clades based on *rpoB* sequences, were picked for draft genome sequencing. For genomic DNA extraction, *Streptomyces* spores from frozen stocks were grown in LB as described above, after which cells were pelleted and genomic DNA was extracted using a Wizard kit (Promega) with added lysozyme and RNase steps, as suggested in the Wizard kit protocol. Genomic DNA from each strain was prepared for sequencing on an Oxford Nanopore Technologies device using sequencing kit LSK109 without shearing. Strains AD2-2 and AD2-3 were sequenced individually, while other strains were multiplexed and sequenced on a MinION system. Bases were called using Guppy v3.2.6 and fast mode. Sequencing statistics are reported in Table 1.

**FIG 1:**
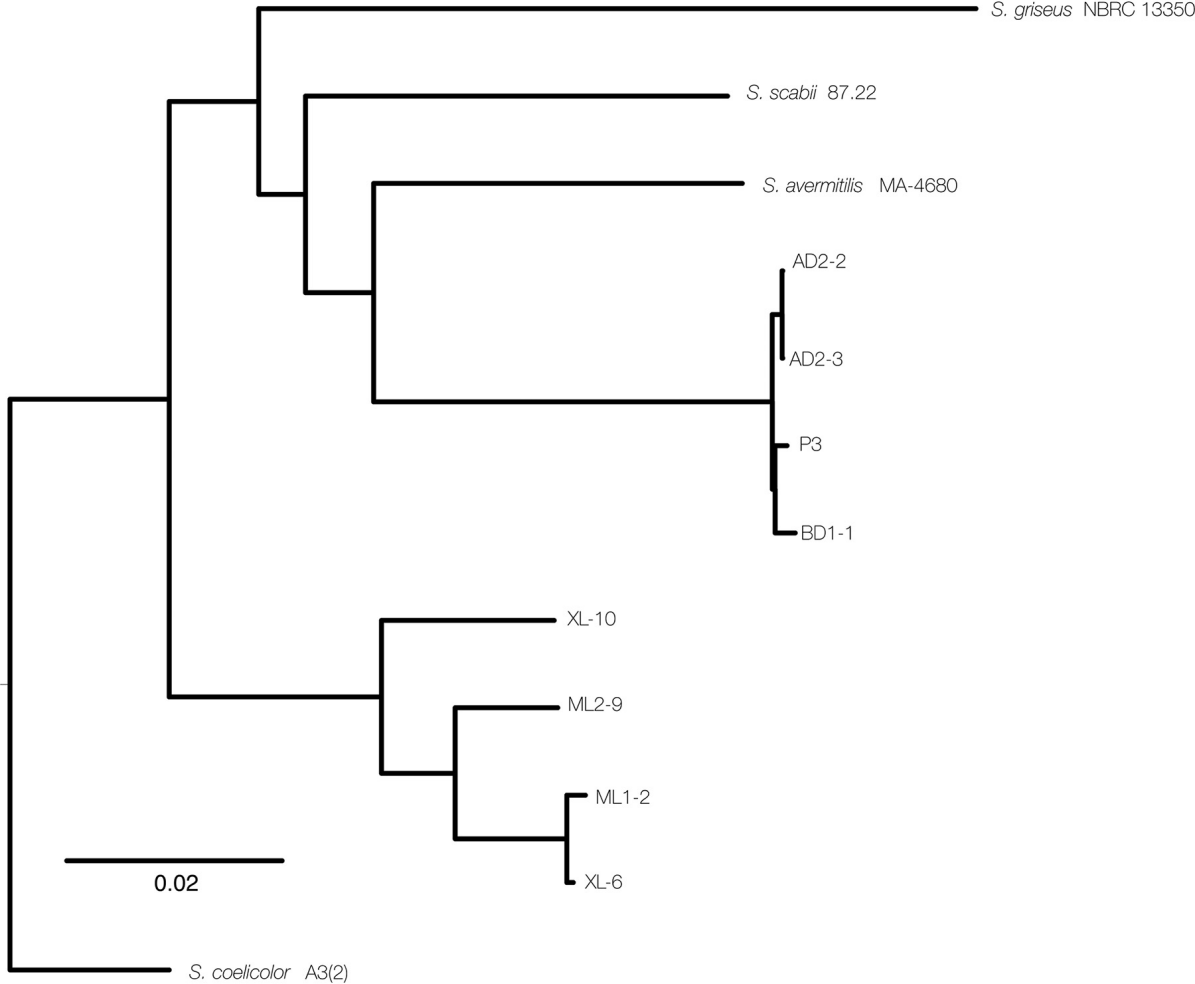
Whole-genome-based phylogeny showing relationships of assemblies and strains reported in this study.

Reads were assembled using Flye v2.7 (6) with default parameters (Table 1). Assemblies were further polished once using a combination of BWA v0.7.17 (7) and Racon v1.4.20 (8). We used RealPHY v1.12 with default parameters (using the assemblies with GenBank accession numbers GCA_000009765.2, GCA_000010605.1, GCA_000091305.1, and GCA_008931305.1 as reference genomes) to explore phylogenetic relationships among Arizona strains using whole-genome information (9) (Fig. 1). According to this inferred phylogeny, the assemblies reported here cluster into two distinct clades (clade 1, ML1-2, ML2-9, XL6, and XL10; clade 2, P3, AD2-2, AD2-3, and BD1-1). Genome assemblies for strains in clade 1 are roughly 7.5 Mb in size, while those in clade 1 are roughly 10 Mb. All strains likely contain plasmids, although the linear topology of *Streptomyces* chromosomes and plasmids makes it difficult to truly characterize assembly structures, given our sequencing strategy.

### Data availability.

All genomic data have been deposited in GenBank under BioProject number PRJNA699332. Genome assembly accession numbers can be found in Table 1.
